# The effect of handling method on the mouse grimace scale in two strains of laboratory mice

**DOI:** 10.1177/0023677215622144

**Published:** 2015-12-10

**Authors:** Amy L Miller, Matthew C Leach

**Affiliations:** School of Agriculture, Food and Rural Development, Agriculture Building, Newcastle University, Newcastle upon Tyne, UK

**Keywords:** refinement, handling, mouse grimace scale

## Abstract

Pain assessment in laboratory animals is an ethical and legal requirement. The mouse grimace scale (MGS) is a new method of pain assessment deemed to be both accurate and reliable, and observers can be rapidly trained to use it. In order for a new pain assessment technique to be effective, we must ensure that the score awarded by the technique is only influenced by pain and not by other husbandry or non-painful but integral aspects of research protocols. Here, we studied 16 male mice, housed under standard laboratory conditions. Eight mice were randomly assigned to tail handling and eight to tube handling on arrival at the unit. On each occasion the mice were removed from their cage for routine husbandry, they were picked up using their assigned handling method. Photographs of the mouse faces were then scored by treatment-blind observers as per the MGS manual (see *Nature Methods* 2010, Vol. 7, pp 447–449), and scores from the two groups were compared. There was no significant difference in MGS scores between the mice that had been handled using a tube compared with the tail. Consequently, these methods of handling did not influence the baseline grimace score given, suggesting that these handling techniques are not confounding factors when establishing baseline MGS scores, further validating this technique.

Millions of mice are used annually in regulated procedures.^1^ An unintended consequence of many of these procedures is likely to be pain, which is a major welfare concern. Prevention or alleviation of such pain is both an ethical and legal requirement, e.g. European Directive EU/2010/63. In order to effectively prevent or alleviate pain, we must have accurate means of pain assessment. Behavioural analysis is the main method used and specific pain behaviours following some procedures have been identified (see example^2^). The mouse grimace scale (MGS) is a new pain assessment method for laboratory mice deemed to be both accurate and reliable, and observers can be rapidly trained to use it.^3^ When considering new pain assessment techniques, we must be confident that any changes we see are indeed pain-related and not an artefact of other integral routine husbandry or research procedures. For example, isoflurane anaesthesia alone has been demonstrated to increase MGS scores in DBA/2 mice,^4^ which must be taken into account if using the MGS for assessment following surgery. Additionally, there is increasing interest in using the MGS for clinical pain assessment, when baseline scores for an individual may not be available as a comparator. Significant variations are found in baseline MGS scores between the sexes and strains,^5^ and the influence of routine husbandry procedures must be established to ensure consistency between baseline scores if this technique is to be used clinically. Any effects of routine handling methods on MGS score have yet to be established.

Handling is likely to be the most common procedure that is experienced by all laboratory mice, as it is integral to carrying out both routine husbandry (e.g. cleaning) and research procedures (e.g. injections). The standard method of handling laboratory mice often involves initially picking mice up by the base of their tails. This method has been shown to result in high levels of anxiety in comparison to initially picking mice up using a familiar tunnel from inside their home cages.^4^ Increased anxiety is a negative reaction both in terms of welfare and scientific validity, with increased numbers of mice required for studies due to increased variability within more anxious groups.

Here, we collected pilot data from two common strains of laboratory mice, CBA and DBA/2, to determine if the handling method (tail versus tube) alone results in changes in MGS scores. If so, any changes in MGS scores related to the handling method would have to be accounted for when using this method of assessment of pain.

Eight CBA and eight DBA/2 male mice (Charles River Laboratories Inc, Kent, UK) weighing 25.6–28.7 g (CBA) and 23.3–26.3 g (DBA/2) at the start of the study were used. Mice were housed in same strain groups of four, in individually-ventilated cages (IVCs) (type 2; Arrowmight, Hereford, UK) with sawdust bedding and nesting material (sizzle nest; Datesand Ltd, Manchester, UK). Environmental enrichment was provided in the form of chew blocks and cardboard tubes (Datesand Ltd, Manchester UK). A seven-day acclimatization period was given prior to the start of the study. The animal room was maintained at 23 ± 1℃, 48% humidity and on a 12/12 h light–dark cycle (lights on at 07:00 h). Food (CRM(P); SDS Ltd, Essex, UK) and tap water were provided ad libitum.

On arrival, one cage of each strain of mice was randomly assigned to ‘tube handling’ and the other to ‘tail handling’. Throughout the study period all interactions with the mice were initiated using the assigned handling technique (i.e. during routine husbandry procedures). The mice from the ‘tail’ group were always initially lifted from their cages using the standard method of securely holding them at the base of their tails. The mice from the ‘tube’ group were always initially lifted from their cages using cardboard tubes that were always present in their home cages in accordance with the method set out by Hurst and West.^6^

Data were recorded in normal mice with no interventions applied other than routine husbandry and handling. Experiments were approved by the Newcastle University’s Animal Welfare and Ethical Review Board. No regulated procedures, which required a Home Office PPL, were carried out as part of this data collection.

Mice were placed individually into small custom-made chambers (80 × 80 × 80 mm), and close-up, high-definition (HD) images of their faces were recorded during a 3 min session. Following filming, the mice were returned to their home cages.

The HD close-up filming was viewed and screen shots were taken on every occasion such that a clear image of the mouse’s face was visible with the exception of when the mouse was grooming. These images were then cropped, leaving only the face of the mouse in the image. Using a random number generator, one image per mouse, per time point was selected. Using the random sequence generator, the selected images were reordered and inserted into a custom-designed Microsoft Excel file. Observers who were blinded to the experimental details, design and purpose scored each photograph using the five facial action units (FAUs) of the MGS as described by Langford et al.^3^ A MGS manual was provided to the scorers for training and reference, but the title of the manual was edited to ‘mouse facial action coding manual’ to limit biasing of scores from the title. Scores for each FAU for every individual photograph were then summed to produce a total MGS score for each image. As multiple individuals scored the images, the mean total score was then calculated.

The MGS scores were compared between tail-handled and tube-handled mice using a Mann–Whitney *U*-test. Results were considered statistically significant when *P* < 0.05.

There was no significant difference in MGS scores between the tail- and tube-handled mice (Figure 1).

**Figure 1.**
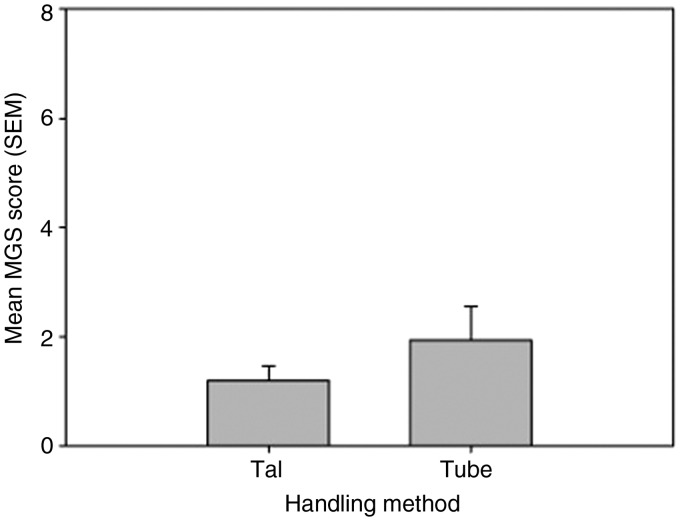
Mean mouse grimace scale (MGS) score (±SEM) for normal laboratory mice, routinely handled by either the tail or using a familiar cardboard tube (*n* = 8/group). Maximum obtainable score is 8.

Effective pain assessment in laboratory mice is critical in terms of both animal welfare and reducing variations within experimental groups. To achieve this, methods of pain assessment must be reliable and must not be influenced by other non-painful but integral aspects of ongoing research procedures, for example routine husbandry procedures or following drug administration.^4^ This will allow baseline MGS scores for a given strain and sex of mouse to be established.^5^ Previous research has demonstrated that mice handled by their tails, rather than using a familiar tube demonstrate increased anxiety.^6^ In the same manner as pain, increasing anxiety in mouse models will lead to increased variability within groups of mice used in research studies. This increase in variation will lead to an increase in the number of mice required to conduct studies. Minimizing anxiety is therefore crucial in terms of welfare and scientific validity. Here, we aimed to determine if handling methods resulted in any changes in MGS scores in normal laboratory mice, as establishing a consistent baseline score is critical for use of the MGS in clinical pain assessment.^5^ Our pilot data assessing the method of handling (i.e. tube versus tail) on the MGS, in normal mice, demonstrated no significant difference in scores given by blinded observers. Consequently, these methods of handling did not influence the grimace score given, suggesting these handling techniques do not influence baseline assessment using the MGS, further validating this technique. Based upon recommendations by Hurst and West,^6^ the practice of tube handling should be observed when handling mice to minimize anxiety and doing so will have no impact on the implementation of the MGS.
